# Continuous flow synthesis of 6-monoamino-6-monodeoxy-β-cyclodextrin

**DOI:** 10.3762/bjoc.19.25

**Published:** 2023-03-09

**Authors:** János Máté Orosz, Dóra Ujj, Petr Kasal, Gábor Benkovics, Erika Bálint

**Affiliations:** 1 Department of Organic Chemistry and Technology, Faculty of Chemical Technology and Biotechnology, Budapest University of Technology and Economics, Műegyetem rkp. 3., H-1111 Budapest, Hungary; 2 Department of Organic Chemistry, Faculty of Science, Charles University, 128 43 Prague 2, Czech Republic

**Keywords:** azidation, continuous flow, β-cyclodextrin, H-cube, 6-monoamino-6-monodeoxy-β-cyclodextrin, monosubstitution, reduction

## Abstract

The first continuous flow method was developed for the synthesis of 6-monoamino-6-monodeoxy-β-cyclodextrin starting from native β-cyclodextrin through three reaction steps, such as monotosylation, azidation and reduction. All reaction steps were studied separately and optimized under continuous flow conditions. After the optimization, the reaction steps were coupled in a semi-continuous flow system, since a solvent exchange had to be performed after the tosylation. However, the azidation and the reduction steps were compatible to be coupled in one flow system obtaining 6-monoamino-6-monodeoxy-β-cyclodextrin in a high yield. Our flow method developed is safer and faster than the batch approaches.

## Introduction

Cyclodextrins (CDs), as cyclic oligosaccharides, consist of a macrocyclic ring of glucose subunits linked by α-1,4-glycosidic bonds [1]. They are widely used in pharmaceutical, food and chemical industries, as well as in agriculture. It has been known for decades, that CD complexation can lead to a significant solubility enhancement of poorly water-soluble molecules, and therefore it can enable the biological testing of drugs, which would otherwise not be possible by any other means [2–3].

Monosubstituted CDs contain only one hydroxy group modified with a functional group. In most cases, the preparation of these compounds is based on the use of a limited amount of the reagent. However, due to the very similar reactivity of hydroxy groups, oversubstitution cannot be avoided during the reaction, thus chromatography or crystallization steps are essential for the preparation of pure monofunctionalized CDs. Alternative approaches use sterically hindered reagents, preventing the approach of the second molecule of the reagent to provide higher yields for the monosubstituted compounds [4]. The three different hydroxy groups on the glucose subunits offer three different sites on the CD molecule where the monofunctionalization can occur. Consequently, monosubstituted CDs can be mixtures of three regioisomers.

The number of known monosubstituted CD derivatives is enormous, since monosubstitution was the almost exclusive reaction for the practical production of selectively modified CDs for a long time. From the synthetic point of view, the most important derivatives are those versatile intermediates that can be effectively transformed according to the requirements of the specific application. The modification of a monosubstituted CD with a suitable functional group is an easier process than the optimization of a new monosubstitution reaction on a native CD [5].

Monotosylation of the primary rim of CDs is the most widely used method to obtain C-6 monofunctionalized CDs. Tosyl chloride (TsCl) reacts with α-, β-, and γ-CD in pyridine to give the C-6-monosubstituted product in about 30% yield (for β-CD) [6–7]. The C-6 regioselectivity can be attributed to the inclusion of pyridine into the CD cavity in such a way that it activates only the hydroxy groups on the primary side. Several alternative methods have been developed with the aim of further improving the yield of monotosylation or replacing pyridine with a more user-friendly solvent [8]. Regardless of which strategy is used, the complete conversion of the starting material into the monosubstituted product does not occur, and a mixture of overtosylated products and unreacted starting CD is formed. The target monosulfonated compound is separated by recrystallization from hot water in the case of the β-CD derivative, and by chromatography in the case of α- and γ-CDs.

The larger cavity size of γ-CD is the reason of polysubstitution when TsCl is used [9]. To ensure a better yield of the monosubstituted product, bulkier sulfonyl chloride reagents are used. 2-Naphthalenesulfonyl chloride in pyridine is one common method. However, the concentration of γ-CD must be lower than 20 mM to favor monosubstitution and to ensure the optimal yield (around 30%) after recrystallization from hot water [9]. Sometimes purification using ion exchange column [10] or reversed-phase chromatography has been reported [11]. An even bulkier sulfonyl chloride reagent, specifically 2,4,6-triisopropylbenzenesulfonyl chloride, can also be used [12]. Again, the reaction is performed in pyridine and the desired monosubstituted product is obtained in 69% yield and 98% purity according to the authors.

Many nucleophiles can react with tosylated CDs to give the corresponding C-6-monofunctionalized CDs. However, alkaline bases cannot be used as nucleophiles due to the intramolecular substitution, resulting in a mono-3,6-anhydro product [13]. On the other hand, sodium azide in *N,N*-dimethylformamide (DMF) reacts with mono-6-*O*-tosyl-CDs to give CD monoazides in high yields. The obtained mono(6-azido-6-deoxy)-CDs (N_3_-CDs) are valuable precursors that can be used as starting materials in azide–alkyne click reactions; furthermore, they can be readily reduced to mono(6-amino-6-deoxy)-CDs (NH_2_-CDs) opening the way for the preparation of amine, thioureido or amide-linked CD scaffolds [14]. Several other nucleophiles can react with mono-6-*O*-tosyl-CDs, such as iodide, dithiol, hydroxylamine, alkylamine or polyalkylamine to give iodo- [15], thio- [16], hydroxylamino- [17], or alkylamino-CDs [18], monosubstituted at position C-6. In addition, the tosyl functional group can be oxidized to an aldehyde using a non-nucleophilic base in dimethyl sulfoxide (DMSO) [19]. The monoaldehyde CDs can be further oxidized selectively to afford the corresponding carboxylic acid derivatives [20].

An alternative strategy to overcome the difficulties associated with the preparation of mono-6-*O*-tosyl-CD intermediates is the direct preparation of 6-monoaldehyde-CD with Dess–Martin periodinane in a fairly good yield of 85%, which can be considered the most efficient reaction used so far for the selective monofunctionalization of CDs [1].

Besides traditional synthetic methods, alternative techniques, such as ultrasound and microwave irradiation [21], as well as mechanosynthesis [22–23] for the functionalizations, such as tosylation or azidation of CDs have been also described [24–25].

Continuous flow approaches have already attracted much attention in the oil, plastic, and fine chemical industries [26]. The vision of faster, safer, cheaper, more flexible and robust production also initiated the paradigm shift from batch reactions to continuous flow processes in the pharmaceutical industry [1,27]. During continuous flow reactions, the target molecules can be produced with better purity, selectivity and in higher yields, as well as in consistent quality due to the precise parameter control, low volume ratio and small quantities. The temperature control is simple and toxic or unstable intermediates are easier to handle, making the overall process safer.

To the best of our knowledge, there is no publication on the continuous flow monosubstitution of CD derivatives. In this paper, first we wished to summarize the batch synthesis of 6^A^-*O*-(*p*-toluenesulfonyl)-β-CD, 6^A^-azido-6^A^-deoxy-β-CD, and 6^A^-amino-6^A^-deoxy-β-CD to see how compatible batch methods are with flow synthesis, and then our main aim was to develop continuous flow approaches for the preparation of the mentioned CD derivatives.

## Results and Discussion

### Batch synthesis of 6^A^-*O*-(*p*-toluenesulfonyl)-β-CD (Ts-β-CD, **2**)

There are three standard methods for the preparation of this general and functional CD derivative. Two of them take advantage of the lower aqueous solubility of β-CD (**1**) and Ts-β-CD (**2**) compared to α- and γ-CD analogs. The third method is mainly used for Ts-α-CD and Ts-γ-CD synthesis, but Ts-β-CD (**2**) can also be prepared by this route. However, before discussing these methods in more detail, some issues related to tosylation reagents should be considered.

There are three tosylating agents utilized for the synthesis of Ts-β-CD, *p*-toluenesulfonyl chloride (TsCl) [14,28–29], (*p*-toluenesulfonyl)imidazole (TsIm) [30–31], and *p*-toluenesulfonic anhydride (Ts_2_O) [32]. TsCl is the first choice due to its low price and availability, being a byproduct of saccharin production. The two other reagents are more expensive or can be prepared in the laboratory from TsCl. No significant differences are observed in the product yields concerning the types of the tosylating agents.

As already mentioned above, there are three standard methods for the preparation of Ts-β-CD (**2**). The first and most common method can be called *heterogeneous* approach [28,33–34]. Here, solid TsCl (sufficiently crushed) is added to a β-CD (**1**) aqueous solution and this heterogeneous mixture is stirred for several hours. Then, aqueous NaOH solution is added and the mixture is stirred for another 10–20 minutes. Unreacted TsCl is filtered off and Ts-β-CD, overreacted byproducts, and β-CD are precipitated after neutralization.

The second method could bear the name *homogeneous* approach [8,14,35]. It is very similar to the previous method, however, TsCl dissolved in MeCN is added to the basic β-CD aqueous solution. Turbidity or even slight precipitation is observed during the process. After a few hours, the reaction mixture is filtered and neutralized to induce precipitation.

The third method works with pyridine as a solvent [29,36–37]. In this method, β-CD is dissolved in pyridine and a pyridine solution of TsCl is added dropwise. After a few hours, the solvent is distilled off and the residue is precipitated in acetone in order to obtain solid β-CD compounds.

Two important things need to be mentioned before closing this part. First, the amounts of TsCl range from 0.5 to 9 equiv in the literature and there is no direct correlation between the yield and the amount of TsCl. Usually 1 to 1.3 equiv are sufficient to ensure good yields. However, the yields bring us to the second important point. As already mentioned, the yield of this reaction is not strongly influenced by the amount of TsCl, but by the purification method. If only the precipitation is carried out, then the crude product is always a mixture of Ts-β-CD (**2**), overreacted byproducts, and starting β-CD (**1**). However, in previous studies this has been reported as a clean product with a yield of almost 50% [31,33]. Recrystallization is mandatory to obtain pure Ts-β-CD (**2**) [38–39]. The best results are obtained by crystallization of the crude product from 50% MeOH/water [18], which we adopted for our batch synthesis of Ts-β-CD (**2**). After proper purification, the yield of the desired product **2** was around 25%. Readers can also find more information on problematic *p*-toluenesulfonylation, subsequent azidation and reduction, in a recently published review [5].

It is clear from the already mentioned facts about the synthesis of Ts-β-CD (**2**), that neither of these methods is suitable for the flow chemistry process. Heterogeneous mixtures should be strictly avoided and pyridine is a toxic compound and should not be used in large-scale syntheses or industrial processes.

### Batch synthesis of 6^A^-azido-6^A^-deoxy-β-CD (N_3_-β-CD) (**3**)

Substitution of the *p*-toluenesulfonyl group of Ts-β-CD (**2**) by azide can be carried out in water [40–41], DMF [14,42], or in their combination [43–44] at elevated temperatures. Water is preferred over DMF due to its lower cost and non-toxicity. However, partial hydrolysis of the *p*-toluenesulfonyl group takes place, so the final product is always contaminated with native β-CD (**1**). Despite this, the product mixture after precipitation from acetone was used in the next reaction step and a proper purification of the targeted monosubsituted compound was performed after this last modification. After precipitation, an apparent yield of more than 90% was noted; however, this value did not take into account native β-CD (**1**) as byproduct. On the other hand, if purification by column chromatography or crystallization is also used, yields of 60–80% can be achieved.

Partial hydrolysis can be avoided by using anhydrous DMF and purifying the starting Ts-β-CD (**2**) by crystallization. In this case, a lower amount of NaN_3_ (1–2 equiv) is required and only purification by precipitation from acetone is necessary. In order to prepare N_3_-β-CD (**3**) in batch, we decided to follow the protocol developed by Jicsinszky and Iványi [14], who performed this reaction in anhydrous DMF using 1.1 equiv of NaN_3_ at 110 °C. The product **3** was purified by repeated precipitation from acetone and isolated in a yield of 81%.

### Batch synthesis of 6^A^-amino-6^A^-deoxy-β-CD (NH_2_-β-CD) (**4**)

Again, there are several common methods for the preparation of NH_2_-β-CD (**4**). The most straightforward route is the reaction of Ts-β-CD (**2**) with condensed NH_3_ in dry DMF [45]. However, this reaction leads to a complex mixture and the yield is around 50% after proper purification.

The Staudinger reduction using triphenylphosphine (PPh_3_) and N_3_-β-CD (**3**) in DMF has been the most popular method for the synthesis of NH_2_-β-CD (**4**) since its first publication by Bonnet et al. [46]. This is despite the fact, that PPh_3_ and its oxidized product (triphenylphosphine oxide) form complexes with β-CD derivatives. This complexation creates difficulties in the purification process, which consists mostly of precipitation from acetone or ion-exchange column chromatography. The price of the reagent needs to be also considered in the case of an attempted large-scale synthesis.

The second most used method is the hydrogenation of N_3_-β-CD (**3**) in the presence of Pd/C under a H_2_ atmosphere. In CD chemistry, this method was first described by Petter et al. [8] in the early 1990s. This method is very popular with small-scale syntheses because only gaseous N_2_ is formed as a byproduct and no purification is required when pure N_3_-β-CD (**3**) is used as substrate. However, for large-scale syntheses, mixing hydrogen with the Pd/C catalyst can be dangerous if an inert atmosphere is not properly maintained. In addition, Pd/C is pyrophoric and tends to ignite when it is separated from the reaction mixture by filtration. However, these two drawbacks are not a problem for large-scale flow synthesis when using an H-Cube system with an incorporated electrolytic cell producing H_2_ in situ from ultrapure water [47]. The Pd/C catalyst is placed in a stainless steel cartridge, so it is not necessary to separate it from the solution after the reaction is complete.

Hydrazine hydrate can also be used as a hydrogen source instead of gaseous H_2_, as described by Jicsinszky and Iványi [14], although this method is not so widespread and has limited potential for a large-scale synthesis. As a special feature, the protocol published by Reddy et al. [48] is worth mentioning, who used metallic indium and ammonium chloride for the reduction of N_3_-β-CD.

### Continuous flow synthesis of 6^A^-*O*-(*p*-toluenesulfonyl)-β-CD (**2**)

**Scheme 1:**
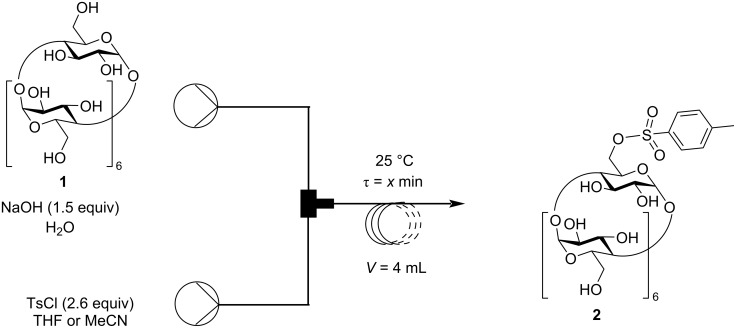
Tosylation of β-CD under continuous flow conditions.

According to the literature, most of the tosylations of CDs took place under heterogeneous conditions. However, small solid particles can easily cause clogging in the narrow tubing of a flow system, therefore homogenous solutions were tried to introduce into the flow systems in all experiments. Regarding the tosylation, β-CD (**1**) and the required base (NaOH) are soluble in water, however, TsCl needs to be dissolved in organic solvents. Alcohols were excluded as possible solvents, as they precipitate β-CD (**1**) and may cause side reactions, but aprotic solvents such as tetrahydrofuran (THF) or acetonitrile (MeCN) were found to be suitable for homogenous conditions, especially a H_2_O/THF 2:1 mixture. This solvent mixture was prepared in situ in the flow tube reactor, as the aqueous solution containing β-CD (**1**) and NaOH (1.5 equiv) was introduced into the reactor at twice the flow rate as the solution of TsCl in THF (Scheme 1).

In this way, twice as many equivalents of TsCl were required compared to the general 1.3 equivalents used in the batch process. The reaction residence time (τ) was varied between 2.2 and 5.3 minutes (Table 1, entries 1–4). The 3 minute residence time was found to be the optimal one and led to a conversion of 24% (Table 1, entry 2). This is a significant advantage compared to batch methods, where similar conversions were achieved in over 2 hours. The flow tosylation was also investigated using acetonitrile instead of THF, however, a much lower conversion was observed (Table 1, entry 4).

**Table 1:** Optimization of the tosylation of β-CD (**1**) under continuous flow conditions.

Entry	Flow rate of TsCl solution [mL/min]	Flow rate of β-CD solution [mL/min]	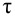 [min]	Solvent	Conversion^a^ [%]

1	0.60	1.20	2.2	H_2_O/THF 2:1	22
2	0.45	0.90	3.0	H_2_O/THF 2:1	24
3	0.25	0.50	5.3	H_2_O/THF 2:1	17
4	0.25	0.50	5.3	H_2_O/MeCN 2:1	11

^a^On the basis of HPLC (214 nm).

### Continuous flow synthesis of 6^A^-azido-6^A^-deoxy-β-CD (**3**)

**Scheme 2:**
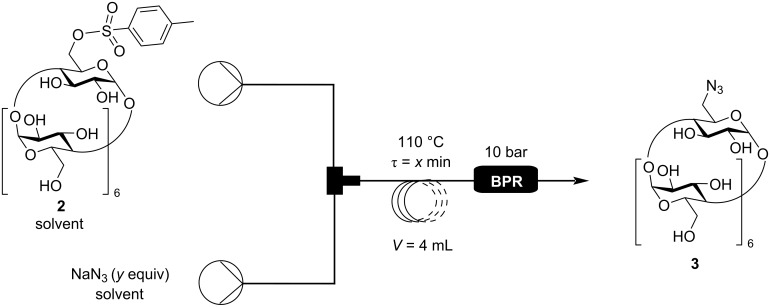
Continuous flow azidation of Ts-β-CD (**2**).

After the tosylation step was successful in flow, next the tosyl–azide substitution was optimized (Scheme 2).

First, the best solvent was sought. Unfortunately, practically no reaction took place when the same solvent was used as for the tosylation reaction (Table 2, entries 1–4), so we had to evaporate the solution exiting the first flow reaction, and change the solvent. According to the literature, DMF proved to be a suitable solvent to conduct this reaction under homogenous conditions. By prolonging the residence time, the conversion greatly increased (Table 2, entries 5–7). However, with longer residence times, increasing hydrolysis of Ts-β-CD (**2**) was observed. To solve this issue, water was excluded from the azidation, which resulted in a total conversion to the corresponding N_3_-β-CD (**3**) (Table 2, entries 8–10). In order allow comparison of the results with those from the batch synthesis, the amount of NaN_3_ was decreased to 1.1 equivalents (Table 2, entries 13–16). Ultimately, the reactor was heated to its limit at 125 °C, by which a satisfying conversion could be achieved. Thus, it can be concluded, that a similar conversion in flow as compared to the batch azidation could be obtained and, in addition as a major advantage, the reaction time was greatly reduced to only 10 minutes.

**Table 2:** Optimization of the continuous flow azidation of Ts-β-CD (**2**).

Entry	NaN_3_ [equiv]	Solvent	*T* [°C]	Total flow rate [mL/min]	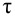 [min]	Conversion^a^ [%]

1^b^	2	H_2_O/THF 2:1	25	1.5	2.7	0
2^c^	2	H_2_O/THF 2:1	25	1.5	2.7	0
3	2	H_2_O/THF 2:1	110	1.5	2.7	0
4	8	H_2_O/THF 2:1	110	1.5	2.7	11
5	8	H_2_O/DMF 1:1	110	0.75	5.3	39
6	8	H_2_O/DMF 1:1	110	0.40	10	45
7	8	H_2_O/DMF 1:1	110	0.20	20	60
8	8	DMF	110	0.40	10	100
9	6	DMF	110	0.40	10	100
10	4	DMF	110	0.40	10	100
11	3	DMF	110	0.40	10	96
12	2	DMF	110	0.40	10	83
13	1.1	DMF	110	0.40	10	45
14	1.1	DMF	125	0.40	10	86
15	1.1	DMF	125	0.20	20	85
16	1.1	DMF	125	0.13	30	78

^a^Based on HPLC (214 nm). ^b^The reaction was carried out using the reaction mixture obtained by the flow tosylation. ^c^Pure starting material was used, which was obtained from batch tosylation.

### Continuous flow synthesis of 6^A^-amino-6^A^-deoxy-β-CD (**4**)

**Scheme 3:**
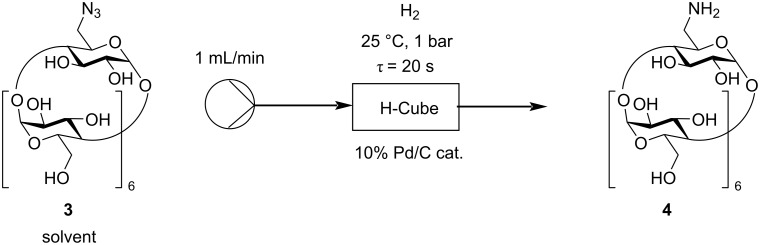
Continuous flow hydrogenation of N_3_-β-CD (**3**).

In the last step of the flow synthesis, the reduction of the N_3_-β-CD (**3**) was investigated in an H-Cube Pro^®^ flow hydrogenating reactor containing a 10% Pd/C pre-packed cartridge (Scheme 3). Generally, a 1 mL/min input flow was used while conducting the hydrogenations during the optimization, which resulted in approximately a 20 second residence time in all cases.

A complete conversion was observed using even the mildest conditions possible (25 °C, 1 bar H_2_ pressure) in aqueous solution (Table 3, entry 1). In spite of this excellent result, the previous azidation reaction proceeded well in DMF, so in order to connect the single flow steps together, the effect of DMF and water/DMF mixtures as solvents had to be studied as well. In DMF, the reaction was not complete and only 30% of NH_2_-β-CD (**4**) were obtained (Table 3, entry 2). However, increasing the ratio of water in the solvent mixture afforded better results (Table 3, entries 3–6). The appropriate ratio of DMF/H_2_O was determined as 1:4, which gave NH_2_-β-CD (**4**) in a yield of 93% (Table 3, entry 5).

**Table 3:** Optimization of the continuous flow hydrogenation of N_3_-β-CD (**3**).

Entry	Solvent	Yield^a^ [%]

1	H_2_O	94
2	DMF	30
3	DMF/H_2_O 1:1	51
4	DMF/H_2_O 1:3	85
5	DMF/H_2_O 1:4	93
6	DMF/H_2_O 1:9	94

^a^Isolated yield.

### Semi-continuous flow system for the synthesis of 6^A^-amino-6^A^-deoxy-β-CD (**4**)

**Scheme 4:**
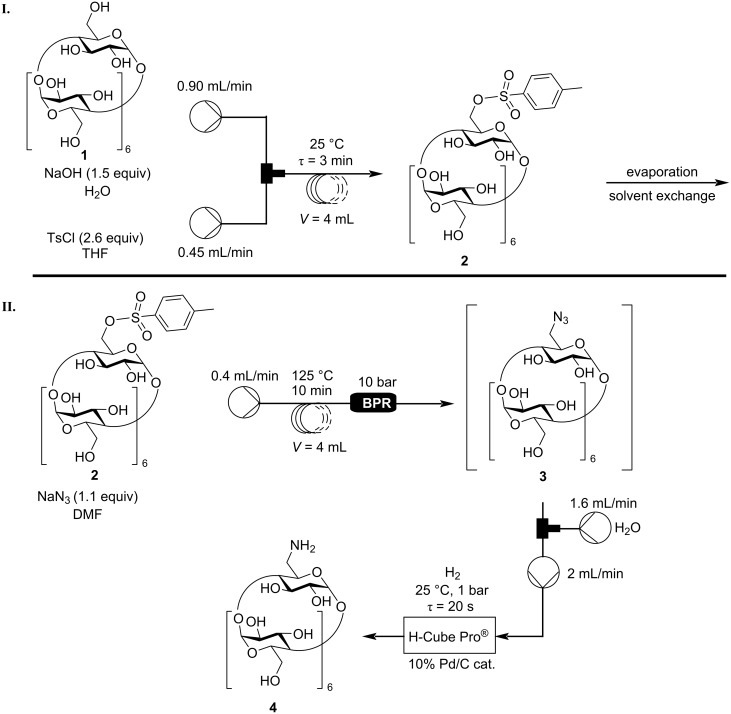
Semi-continuous flow system for the synthesis of NH_2_-β-CD **4**.

The main goal of this part of the work was to establish a continuous flow method for the synthesis of NH_2_-β-CD (**4**) from β-CD (**1**). Thus, after the optimization of the single reaction steps, the connection of these steps was remaining. First of all, it was concluded, that the initial tosylation step could not be connected to the azidation step since in the tosylation’s optimal solvent, the azidation did not proceed and vice versa (Table 4). The only solution was to conduct the tosylation under flow conditions separately from the other two steps (Scheme 4I), evaporate the water/THF solvent, and introduce the Ts-β-CD (**2**) dissolved in DMF to the second part of the flow system. In order to simplify the azidation and subsequent reduction, Ts-β-CD (**2**) prepared from batch and properly purified was utilized.

**Table 4:** Optimal reaction conditions for each flow reaction step.

Synthesis step	Reagent	Solvent	*T* [°C]	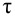 [min]	Conversion^a^ [%]

tosylation	2.6 equiv TsCl	H_2_O/THF 2:1	25	3	20
azidation	1.1 equiv NaN_3_	DMF	125	10	81
hydrogenation^b^	H_2_	H_2_O/DMF 1:4	25	0.3	93

^a^Isolated yield. ^b^1 bar H_2_ pressure and 10 mol % Pd/C catalyst were used.

The azidation and the hydrogenation were compatible with each other, however, after the azidation took place, water needed to be introduced to the system before the hydrogenation to ensure full conversion during the reduction. According to our previous results, the reduction of N_3_-β-CD (**3**) went to completion in a DMF/H_2_O 1:4 mixture, so this solvent was chosen for the hydrogenation step in order to avoid re-optimization. This way, the exiting solution of 0.4 mL/min from the azidation reaction was joined with a 1.6 mL/min flow of water, and this new solution was gathered in a buffer container, from where the reaction mixture was immediately forwarded to the H-Cube Pro^®^ (Scheme 4II). Although the hydrogenation was optimized for a 1 mL/min input flow rate, the catalyst cartridge was changed to another one, which doubled the reaction volume. In conclusion, a 2 mL/min input flow rate required no re-optimization, as the residence time remained the same. Under these conditions, the connected azidation and reduction steps led to an almost complete conversion of Ts-β-CD (**2**). The output of the H-Cube Pro^®^ was partially evaporated, the product **4** precipitated with acetone and isolated in a yield of 91%.

## Conclusion

In conclusion, we have developed continuous flow methods for the monotosylation of β-CD (**1**), for the azidation of 6^A^-*O*-(*p*-toluenesulfonyl)-β-CD (**2**), and for the reduction of 6^A^-azido-6^A^-deoxy-β-CD (**3**). The flow methods are novel approaches for the preparation of the target compounds and were optimized for each case. Comparing the flow processes with batch methods, it can be concluded that similar yields were obtained in both cases, however, under continuous flow conditions, the reaction time could be reduced from hours to minutes. Finally, we made an attempt to connect the three reaction steps with each other in a continuous flow system. It was found that a solvent exchange step was required after the tosylation, however, the azidation and the reduction steps were compatible to be coupled in one flow system. Using our semi-flow method developed, the production of 6^A^-amino-6^A^-deoxy-β-CD (**4**) could be carried out in a safer way due to the easier handling of toxic derivatives and with more precise parameter control. Moreover, the reactions can be performed within a much shorter reaction time than under batch conditions.

## Supporting Information

Experimental procedures, characterization data, details of the NMR structural determination of the products and copies of ^1^H NMR spectra for the compounds synthesized.

File 1 Experimental section and copies of NMR spectra.
