# Supplementary material for: Continuous flow synthesis of 6-monoamino-6-monodeoxy-β-cyclodextrin
Source: Beilstein J Org Chem. 2023 Mar 9;19:294–302. doi: 10.3762/bjoc.19.25 (PMC10012048; doi:10.3762/bjoc.19.25)
Supplement: File 1 — Experimental section and copies of NMR spectra. [file Beilstein_J_Org_Chem-19-294-s001.pdf]

BEILSTEIN JOURNAL OF ORGANIC CHEMISTRY

Supporting Information

for

Continuous flow synthesis of 6-monoamino-6-monodeoxy-β-cyclodextrin

János Máté Orosz, Dóra Ujj, Petr Kasal, Gábor Benkovics and Erika Bálint

Beilstein J. Org. Chem. **2023,** *19,* 294–302. doi:10.3762/bjoc.19.25

Experimental section and copies of NMR spectra

License and Terms: This is a supporting information file under the terms of the Creative Commons Attribution License (https://creativecommons.org/licenses/by/4.0). Please note that the reuse, redistribution and reproduction in particular requires that the author(s) and source are credited and that individual graphics may be subject to special legal provisions.
The license is subject to the *Beilstein Journal of Organic Chemistry* terms and conditions: (https://www.beilstein-journals.org/bjoc/terms)

Table of contents

General information, instruments, and materials S1

Calculation of the conversion.. S3

General procedure for the batch synthesis of 6^A-*O*-*p*-toluenesulfonyl-β-CD (**1**) S6

General procedure for the batch synthesis of 6^A-azido-6^A-deoxy-β-CD (**2**) S6

General procedure for the flow synthesis of 6^A-*O*-*p*-toluenesulfonyl-β-CD (**2**).......... S6

General procedure for the flow synthesis of 6^A-azido-6^A-deoxy-β-CD (**3**)................ S7

General procedure for the flow synthesis of 6^A-amino-6^A-deoxy-β-CD (**4**) S7

General procedure for the flow synthesis of 6^A-amino-6^A-deoxy-β-CD (**4**) from 6^A-*O*-*p*-toluenesulfonyl-β-CD (**2**)... S8

^{1}H NMR spectra.. S9

References S12

General information, instruments, and materials

Deionized H_2O was utilized for reactions. Native β-CD was purchased from Cyclolab Kft. (Hungary). Other reagents were purchased from common commercial sources (Merck, Lach-Ner) and used without further purification. The laboratory glassware was dried at 150 °C in an oven before use if the reaction required dry conditions.

The flow tosylation and flow azidation took place in an Asia® heated tube reactor (V = 4 mL) made by Syrris Ltd. (Figure S1), the flow hydrogenation was conducted in the H-Cube Pro® reactor, purchased from ThalesNano Inc (Figure S2). In the case of flow tosylation and flow azidation Asia® syringe pumps were used to introduce the solutions to the corresponding flow systems. If not stated otherwise, 250 µL and 500 µL Asia® syringes were used. In the case of flow hydrogenation, an HPLC pump made by ThalesNano Inc. was used to introduce the starting material into the reactor. Polyether ether ketone (PEEK) tubing (1.6 mm in diameter) was utilized as connections between the Asia® pumps and the Asia® reactor modules. If not stated otherwise, the reactors and the tubings were washed with the reactions' corresponding solvents at 1 mL/min flow rate before and after synthesis for 10 minutes.

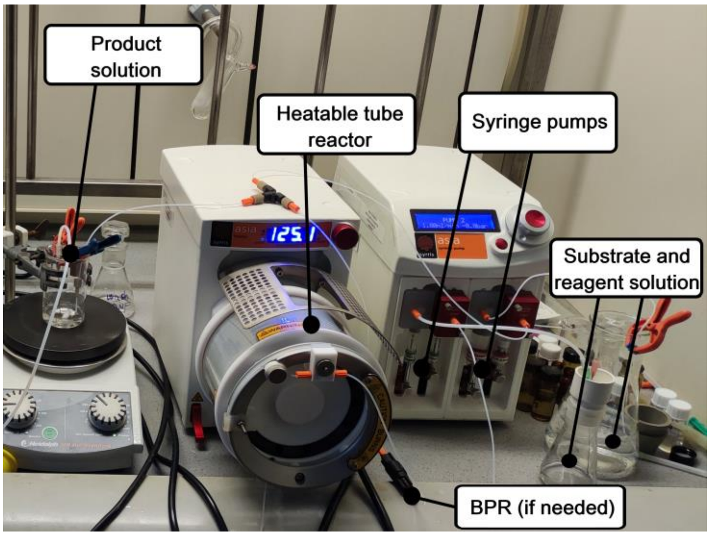

Figure S1: Asia® Heater module and syringe pumps for tosylation and azidation.

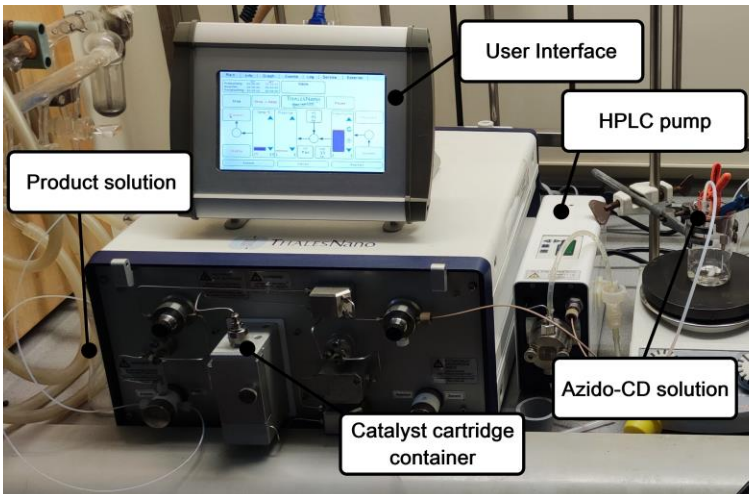

Figure S2: H-Cube Pro® hydrogenation flow reactor for the synthesis of 6^A-amino-6^A-deoxy-β-CD (**4**).

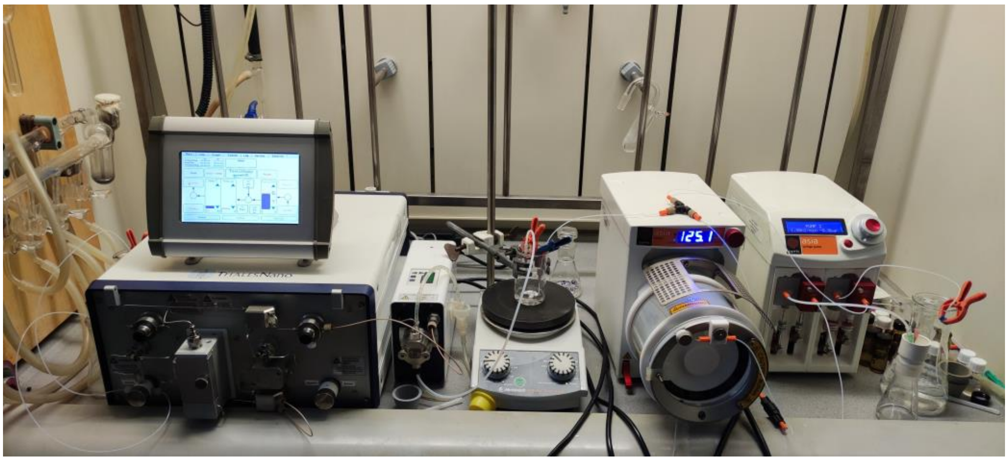

Figure S3: Continuous flow system for the synthesis of 6^A-amino-6^A-deoxy-β-CD (**4**) from 6^A-*O*-*p*-toluenesulfonyl-β-CD (**2**).

Thin layer chromatography (TLC) was performed on aluminium sheets pre-coated with a layer of silica gel 60 F254, purchased from Merck. The plates were developed in a saturated chamber; the mobile phases are given at each procedure in volume/volume ratio.

Spots on TLC plates were detected by using several different methods:

- M1 = a UV lamp (λ = 254 nm).
- M2 = dipping the TLC plate in a 50% w/w H_2SO_4 aq. solution, followed by heating to 250 °C by a heat gun.

HPLC measurements were carried out on an Agilent 1100 HPLC system (Agilent, Waldbronn, Germany) equipped with UV–vis detector.

The following chromatographic conditions were applied for the analysis of 6^A-O-*p*-toluenesulfonyl-β-CD and 6^A-azido-6^A-deoxy-β-CD compounds: Inertsil ODS-3 column (150 mm × 4.6 mm, 3 µm; GL Sciences, Nakano-ku, Tokyo, Japan). Eluents: mobile phase A: purified water, mobile phase B: acetonitrile. Gradient program: 0 min–0% B, 10 min–40% B, 12 min–100% B. Run time was 15 min, the equilibration time was 5 min. The flow rate was 0.8 mL/min, the column thermostat was set at 30 °C and the injection volume was 10 µL. The UV detection was performed at 214 nm.

High resolution mass spectrometric measurements were performed using a Sciex 5600+ Q-TOF mass spectrometer in positive electrospray ionization mode.

The ^{1}H NMR spectra were acquired on a Bruker AVANCE II spectrometer operating at 500 MHz. Samples were dissolved in DMSO-d_6. Chemical shifts are given in ppm; coupling constants *J* are given in Hz.

Calculation of the conversion

The conversion for all samples was calculated by a calibration curve.

Preparation of the calibration samples:

Stock solutions containing 4 mg/mL 6^A-O-*p*-toluenesulfonyl-β-CD (**2**) and 6^A-azido-6^A-deoxy-β-CD (**3**), respectively, were prepared from solid reference samples with purified water and then diluted 10-, 5-, 2.5-, 1.7- and 1-fold with purified water. The samples obtained from the continuous flow synthesis were analyzed after the appropriate dilution with purified water, to meet the linearity criteria of the method.

An example chromatogram of a sample from the continuous flow synthesis and an example of the calibration (chromatograms, calibration curve and details) are shown in Figures S4–S6.

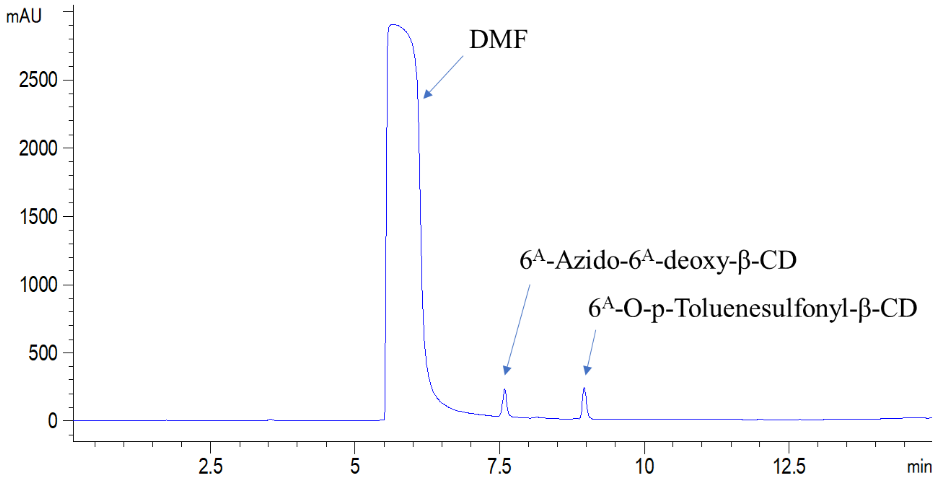

Figure S4: Chromatogram of a sample from the continuous flow synthesis measured by the above detailed HPLC method.

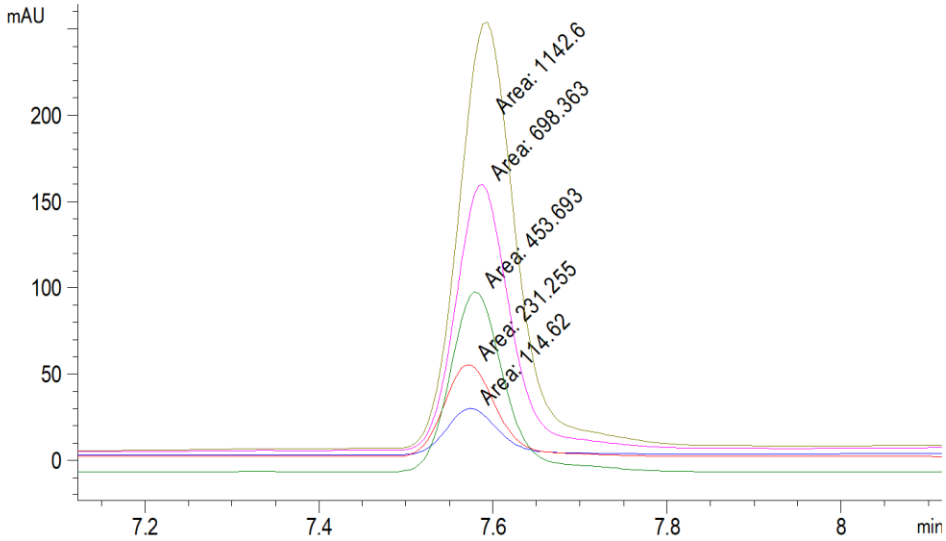

Figure S5: Overlaid chromatograms of the 6^A-azido-6^A-deoxy-β-CD (**3**) calibration samples measured by the above detailed HPLC method.

Stock solution:	21.2 mg / 5mL	4.24	mg/mL
Sample	Dilution	c / (mg/mL)	Area
CAL_1	10	0.424	114.6
CAL_2	5	0.848	231.3
CAL_3	2.5	1.696	453.7
CAL_4	1.7	2.494	698.4
CAL_5	1	4.240	1142.6

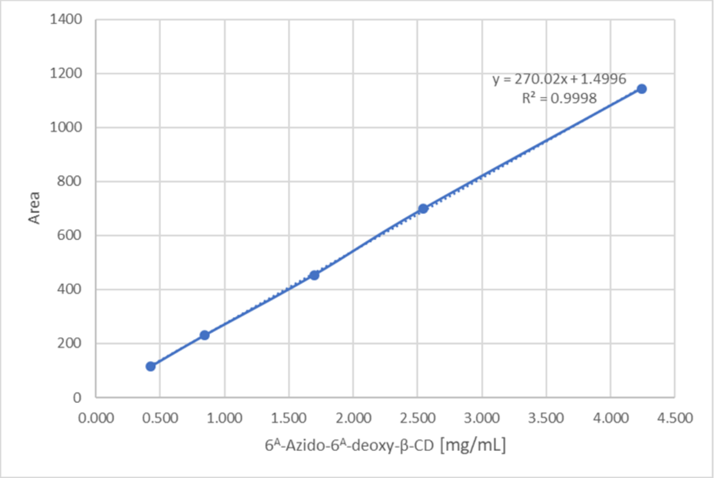

Figure S6: Details of the calibration and the calibration curve.

General procedure for the batch synthesis of 6^A-*O*-*p*-toluenesulfonyl-β-CD (2)

The compound was prepared according to the previously published procedure [S1]. The suspension of β-CD (10.0 g, 8.8 mmol, dried at 70 °C for 1 hour using a membrane pump) and powdered TsCl (2.5 g, 13.2 mmol) in H_2O (200 mL) was stirred for 2 hours at room temperature. A solution of NaOH (4.2 g) in H_2O (40 mL) was added. After 10 minutes, unreacted TsCl was separated by filtration, the filtrate was neutralized with 10 M HCl, and the solution was put into a fridge for a night. The resulting precipitate was collected by filtration, washed with ice cold H_2O, and dried at 80 °C using a membrane pump. The crude product was purified by repeated recrystallization from H_2O/MeOH 1:1 mixture. The crystallization purification was monitored by TLC using iPrOH/H_2O/EtOAc/conc. NH_3 aq. solution 6:3:1:1 mixture. The pure product was dried at 80 °C using a membrane pump and obtained as a white crystalline solid in a yield of 23% (2.6 g).

General procedure for the batch synthesis of 6^A-azido-6^A-deoxy-β-CD (3)

The compound was prepared according to the previously published procedure [S2]. In a Schlenk flask, 6^A-*O*-*p*-toluenesulfonyl-β-CD (**2**, 1.0 g, 0.8 mmol, dried at 70 °C for 1 hour using a membrane pump) was dissolved in dry DMF (5 mL) and NaN_3 (0.06 g, 0.9 mmol) was added. The mixture was heated to 110 °C and stirred for 2 hours. The reaction progress was monitored by TLC using iPrOH/H_2O/EtOAc/conc. NH_3 aq. solution 6:3:1:1 mixture. The reaction mixture was poured into acetone (20 mL), the resulting precipitate was isolated by filtration, and the solid was washed with acetone (3 × 10 mL). The solid (2.3 g) was dried at 80 °C for 2 hours using a membrane pump. The crude product (1 g) was dissolved in hot H_2O (5 mL) and poured into acetone (25 mL). The precipitated product was filtered and washed with acetone (3 × 10 mL). The product was dried at 80 °C using a membrane pump and obtained as a white solid in a yield of 81% (0.75 g).

General procedure for the flow synthesis of 6^A-*O*-*p*-toluenesulfonyl-β-CD (2)

Native β-cyclodextrin (**1**, 220 mg, 0.19 mmol) and solid NaOH (12 mg, 0.29 mmol) were dissolved in 5 mL distilled water (solution A). Tosyl chloride (96 mg, 0.50 mmol) was dissolved in 2.5 mL tetrahydrofuran (THF) (solution B). The Asia® syringe pumps were set to 900 µL/min (for solution A) and 450 µL/min (for solution B) flow rates. The pumps were started, and no product was collected in the first 3.5 minutes. After this,

the product was collected for 2 minutes. Finally, for another 3.5 minutes a water/THF 2:1 mixture was introduced to the system at the same flow rates, while the product was still collected. After that THF was evaporated from the collected mixture. Unreacted TsCl was separated by filtration, the filtrate was neutralized with 10 M HCl, and the solution was put into a fridge overnight. The resulting precipitate was collected by filtration, washed with ice cold H_2O, and dried at 80 °C using a membrane pump. The crude product was purified by repeated recrystallization from a H_2O/MeOH 1:1 mixture. The crystallization purification was monitored by TLC using iPrOH/H_2O/EtOAc/conc. NH_3 aq. solution 6:3:1:1 mixture. The pure product was dried at 80 °C using a membrane pump and obtained as a white crystalline solid in an 20% yield (0.049 g).

General procedure for the flow synthesis of 6^A-azido-6^A-deoxy-β-CD (3)

6^A-*O*-*p*-Toluenesulfonyl-β-CD (**2**, 100 mg, 0.08 mmol) and 0.09 mmol of NaN_3 (5.5 mg) were dissolved in 2 mL *N,N*-dimethylformamide (DMF). The Asia® syringe pump was set to 400 µL/min flow rate, and the Asia® heater module was set to 125 °C. When the temperature in the tube reactor reached the set temperature, the pumps were started, and for 5 minutes the solution containing the cyclodextrin and the azide was introduced to the system. After 5 minutes, neat DMF was introduced to the system with the same flow rate for 7.5 minutes. After this, the product was collected for 5 minutes. The reaction mixture was poured into acetone (2 mL), the resulting precipitate was isolated by filtration, and the solid was washed with acetone (3 × 1 mL). The solid was dried at 80 °C for 2 hours using a membrane pump. The crude product was dissolved in hot H_2O (1 mL) and poured into acetone (3 mL). The precipitated product was filtered and washed with acetone (3 × 1 mL). The product was dried at 80 °C using a membrane pump and obtained as a white solid in a yield of 81% (0.075 g).

General procedure for the flow synthesis of 6^A-amino-6^A-deoxy-β-CD (4)

6^A-Azido-6^A-deoxy-β-CD (**3**, 100 mg, 0.09 mmol) was dissolved in 10 mL DMF/water 1:4 mixture. A 30 × 4 mm 10% Pd/C CatCart® catalyst cartridge was placed into the H-Cube Pro® reactor. The temperature was set to 25 °C, the H_2 pressure was set to 1 bar. The HPLC pump of the H-Cube Pro® reactor was set to 1 mL/min flow rate. The HPLC pump was started and in the first 4 minutes, no product was collected.

After 4 minutes, the product was collected for 6 minutes. When the solution containing the starting material ran out, DMF/water 1:4 mixture was pumped through the system for additional 4 minutes. The product solution was evaporated, and acetone was added. The precipitated product was filtered, dried and obtained as white solid. Yield: 93% (0.095 g).

General procedure for the continuous flow synthesis of 6^A-amino-6^A-deoxy-β-CD (4) from 6^A-*O*-*p*-toluenesulfonyl-β-CD (2)

6^A-*O*-*p*-Toluenesulfonyl-β-CD (**2**, 1.00 g, 0.78 mmol) and NaN_3 (55 mg, 0.85 mmol) were dissolved in 20 mL DMF. A 70 × 4 mm 10% Pd/C CatCart® catalyst cartridge was placed into the H-Cube Pro® reactor. One pair of the Asia syringes was changed to 2.5 mL and 5 mL syringes for water input. The 0.25 mL and 0.5 mL syringe pumps were set to a 400 µL/min flow rate. For 12 minutes, the DMF solution was pumped through the reactor. After that, distilled water was also pumped into the system at a 1.6 mL/min flow rate. The water flow evaded the tube reactor, and was mixed with the reaction mixture after the tube reactor. The water/DMF mixture was collected in a buffer container for 10 minutes. After this, the H-Cube Pro® reactor was set to 25 °C temperature and 1 bar H_2 pressure. The HPLC pump of the H-Cube Pro® reactor was set to 2 mL/min flow rate, and continuous hydrogenation took place. The DMF solution of the starting material was introduced to the system for another 28 minutes, and when the starting solution was depleted, neat DMF was introduced to the Asia® system for 12 minutes at the same flow rate as before. After this, both Asia® pumps were stopped, and the hydrogenation was continued until the water/DMF reaction mixture was depleted (10 minutes) from the buffer container. The product solution was evaporated to 100 mL volume, and acetone (500 mL) was added. The precipitated product was filtered, dried and obtained as white solid. Yield: 91% (0.92 g).

Copies of ^{1}H NMR spectra

6^{A}-*O*-(*p*-Toluenesulfonyl)-β-CD (2) (DMSO-d_6, 500 MHz)

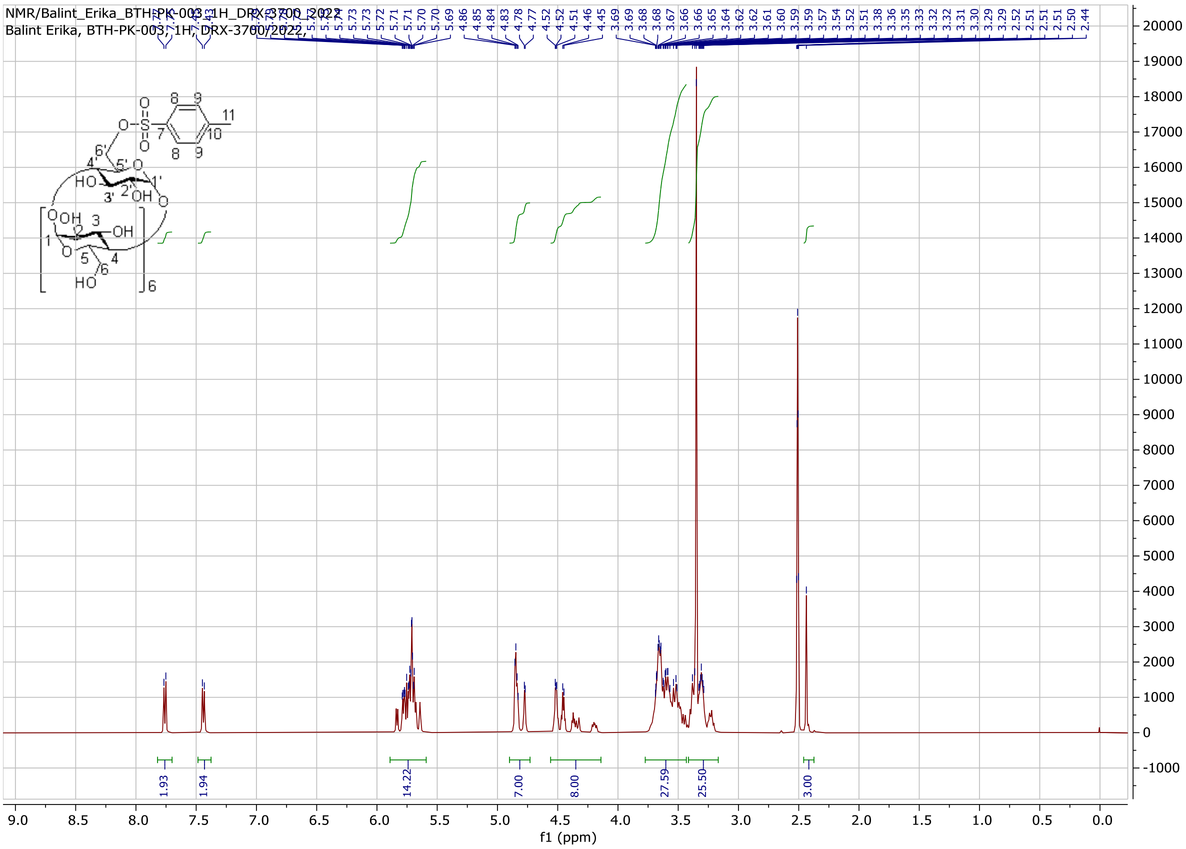

6^{A}-*O*-*p*-Toluenesulfonyl-β-CD (2)

^{1}H NMR (DMSO-d_6) δ 2.44 (s, 3H), 3.19–3.75 (m, 40H, solvent overlay), 4.18–4.53 (m, 8H), 4.75–4.83 (m, 7H), 5.62–5.79 (m, 14H), 7.44 (d, J = 8.2 Hz, 2H), 7.76 (d, J = 8.2 Hz, 2H). ^{1}H NMR spectrum is in accordance with the literature [S1].

$[M + H]^{+}_{found}$ = 1289.3853, $C_{49}H_{77}O_{37}S$ requires 1289.3859.

6^A-Azido-6^A-deoxy-β-CD (3) (D_2O, 500 MHz)

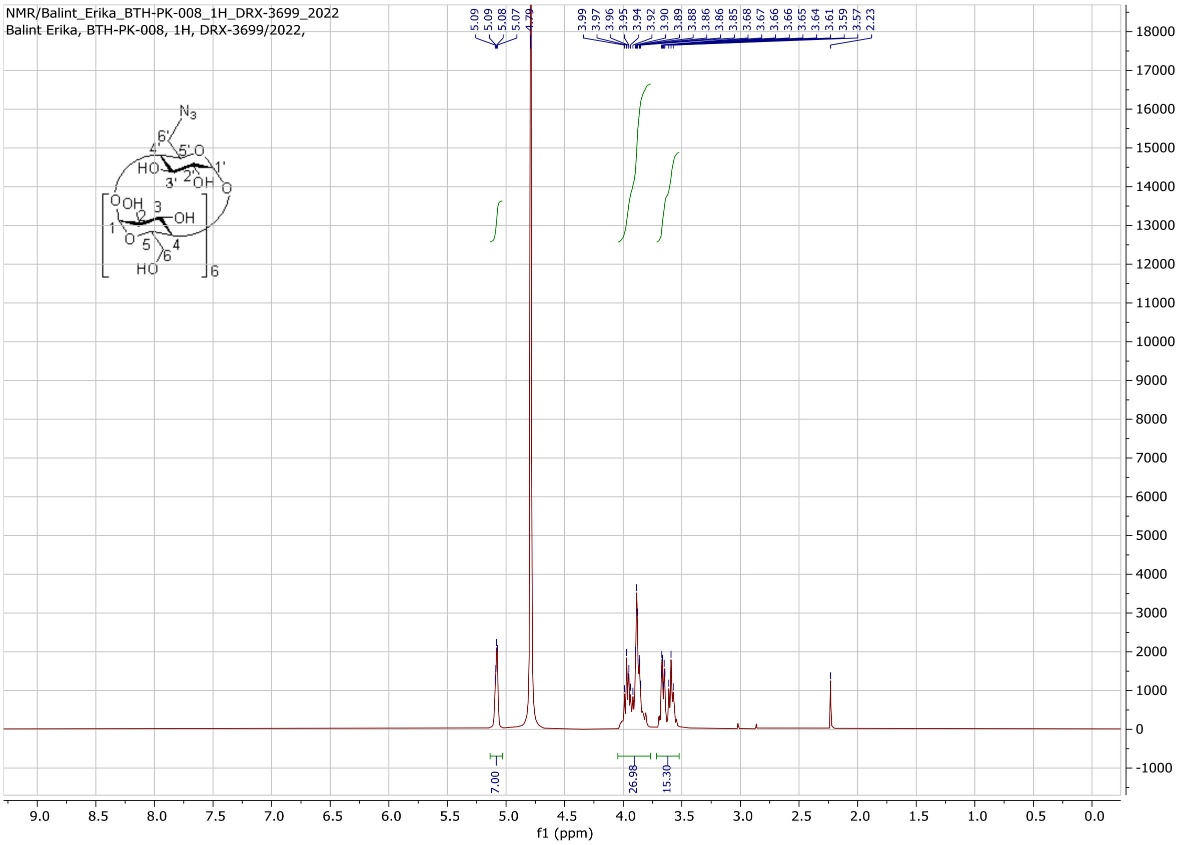

6^A-Azido-6^A-deoxy-β-CD (3)

^1H NMR (D_2O) δ 3.54–3.70 (m, 15H), 3.80–4.02 (m, 27H), 5.06–5.10 (m, 7H). ^1H NMR spectrum is in accordance with the literature [S3].

$[M + H]^+_{found}$ = 1160.3802, $C_{42}H_{70}N_3O_{34}$ requires 1160.3835.

6^{A}-Amino-6^{A}-deoxy-β-CD (4) (D_2O, 500 MHz)

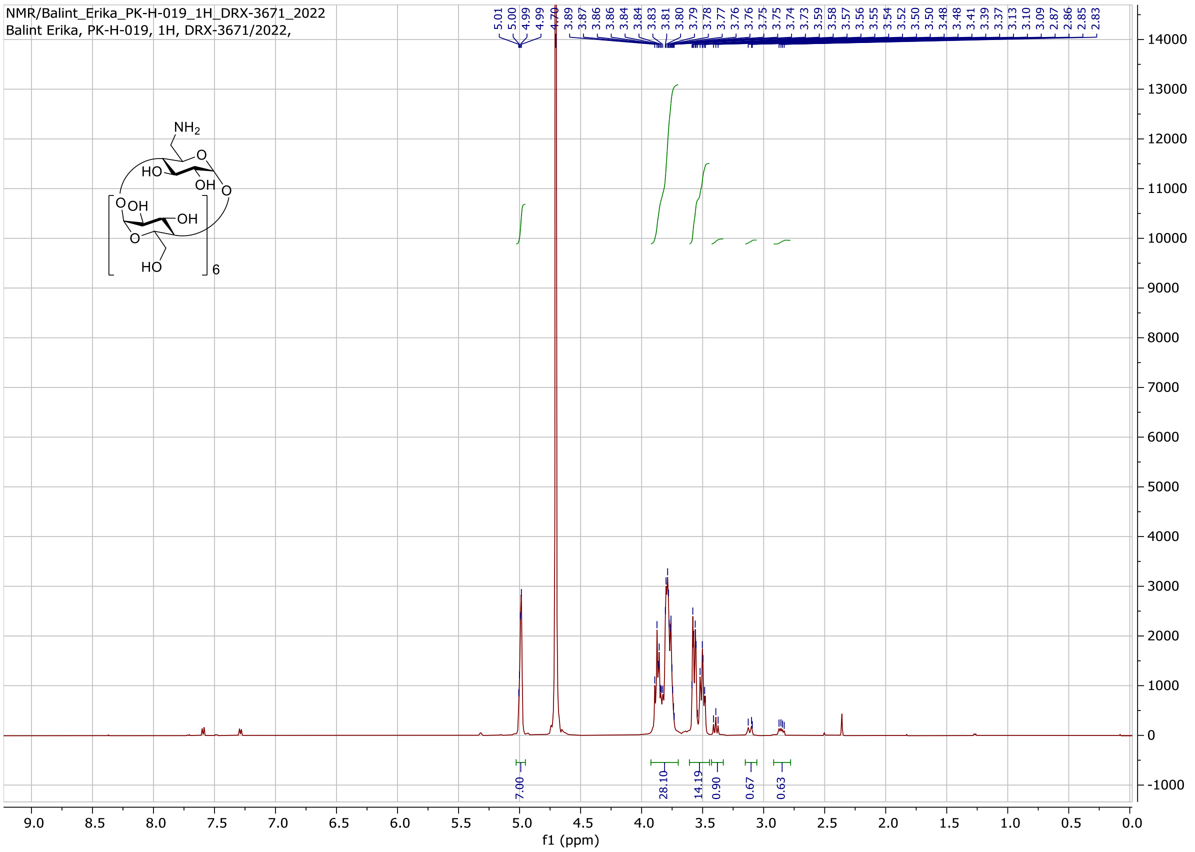

6^{A}-Amino-6^{A}-deoxy-β-CD (4)

^{1}H NMR (D_2O) δ 2.82–2.89 (m, 1H), 3.09 (d, J = 13.6 Hz, 1H), 3.39 (t, J = 9.4 Hz, 1H), 3.46–3.690 (m, 14H), 3.70–3.94 (m, 28H), 4-95–5.03 (m, 7H). ^{1}H NMR spectrum is in accordance with the literature [S4].

$[M + H]^{+}_{found}$ = 1134.3925, $C_{42}H_{72}NO_{34}$ requires 1134.3936.

References

S1. Popr, M.; Hybelbauerova, S.; Jindrich J. *Beilstein J. Org. Chem.* **2014**, *10*, 1390–1396. doi: 10.3762/bjoc.10.142

S2. Jicsinszky, L.; Ivanyi, R. *Carbohydr. Polym.* **2001**, *45*, 139–145. doi: 10.1016/S0144-8617(00)00319-2

S3. Ji, J.; Wu, W.; Wei, X.; Rao, M.; Zhou, D.; Cheng, G.; Gong, Q.; Luo, K.; Yang, C. *Chem. Commun.* **2020**, *56*, 6197–6200. 10.1039/D0CC02055A

S4. Puglisi, A.; Spencer, J.; Clarke, J.; Milton, J. *J. Incl. Phenom. Macrocycl. Chem.* **2012**, *73*, 475–478. doi: 10.1007/s10847-011-0054-z
